# Pathology and Treatment of Alveolar Abscess

**Published:** 1910-11-15

**Authors:** Stewart L. McCurdy

**Affiliations:** Pittsburg, Pa.


### PATHOLOGY AND TREATMENT OF ALVEOLAR ABSCESS.

### AND A PLASTIC OPERATION TO CLOSE NASO-ORAL FISTULA AND A NEW OPERATION FOR MANDIBULAR NECROSIS.*

STEWART L. McCURDY, M.D., PITTSBURG, PA.

The capital operation of dentistry is devitalizing pulp and filling of the root canals, as an abdominal section or the opening of an important joint is the capital operation of surgery.

If the peritoneal cavity or synovial membrane is infected during an operation, serious and dangerous inflammatory processes follow; infections of the former in many instances result in death, and infections of the latter result in ankylosis

1. A measure which has proved useful in bony suppurations and alveolitis is the internal administration of ethereal tincture of phosphorus (1 to 1,000) 3 minims after each meal, well diluted with water. If much pyorrhea infection be present, but the teeth sound, the above remedy followed by solution of mercuric chlorid (1 to 1,000 three minims between meals, is worthy of more trial. At the same time the gums should be painted with Dr. E. S. Talbot's iodin and glycerin mixture, or twice daily, anteriorly and posteriorly, with a dilution of iodin liniment (B. P.) made by adding to 2 drams of liniment water to make 4 ounces. In cases of adults in whom there is much gastric flatulence and pain showing intestinal autointoxication, as well as in the infantile form of digestive disturbance, silicate of sodium acts nicely as a sedative and antiferment. Sodium silicate 172 gm., dissolved in 1 liter of water, to which a trace of fluosilicate of magnesia and carbonate of lithium has been added, may be given in doses of one to two teaspoonfulls three times daily before food, according to age and conditions present. Where the teeth are loose and very sensitive protargol in glycerole, used alone or with an electrode on an interrupted current, helps.

*Read in the Section on Stomatology of the American Medical Association, at the Sixty-first Annual Session, at St. Louis, June, 1910.

and even amputation. It is possible for the general surgeon to perform the operations without mortality only because of the great care exercised by him preparatory to and during the operation. On the other hand, the most delicate operation in dentistry, requiring the most technical skill as well as demanding perfect asepsis in its practice, is the devitalizing and extracting of pulps and filling these cavities. If infection occurs on account of carelessness on the part of the dentist, the too common alveolar abscess is the result. Alveolar abscess, or the so-called pus sack so frequently found on the apex of a tooth after extraction, is the forerunner of 90 per cent. of all the destructive diseases of the maxillary bones.

### SERIOUS CONSEQUENCES OF ALVEOLAR ABSCESS.

The pathologic changes which occur in the so-called alveolar abscess begin at the apex of the root of a tooth. The first change is quite small, beginning in the form of an infiltrate, which later liquifies. This change involves the tissues immediately around the apex of the root either destroying or promoting the absorption of the bone. The process of the destruction is in the direction of the least resistance from the root involved, which experience demonstrates to be smaller on the buccal side of the alveolar process. The destruction continues to the surface of the bone when the external manifestation of the abscess is present, namely, a fluctuating tumefaction on the external surface of the bone. This continues to destroy tissue in proportion to its activity, which depends on the variety of germ responsible for the disease.

The symptoms are well known to every dentist. Pain is in proportion to the rapidity of the course and, as a rule, is not prominent, a slight ache of the face being the most common characteristics.

If the root of the tooth affected is in proximity to the maxillary sinus, this cavity will be infected, resulting in antral disease. Occasionally when the disease is in the mandible in proximity to the central canal a great portion of the bone will become involved before the surface is reached and the ordinary fluctuating cyst described above is not found.

In many of these abscesses the bony floor of the antrum is destroyed, yet the antral cavity is not entered because the membranous floor is still intact. If the distention of the abscess cavity is great or if it continues for a considerable length of time, the nasal or antral cavity will be entered and in the former instance the antrum becomes infected, and in the latter instance a nasal fistula established.

When a fistulous opening is established from one of these abscesses, the orifice may be through the alveolus, the external or internal surface of the bone. In cases of fistulas communicating with a cavity in which there was the root of a tooth denuded of its periosteum and apical circulation, I have not seen the opening close without the extraction of the tooth. This applies to chronic openings persisting for three or more months.

An alveolar abscess of the mandible is rather common. It is usual, also, for the abscess to break through the cheek rather than into the oral cavity. It is all a question of dependent drainage. The reason that alveolar abscesses of the superior bone repair better than those below is that the drainage above is superior.

The second serious consequence of alveolar abscess is a more grave variety of destruction of the maxillary bone, when the nasal floor, membranous and osseous, is destroyed, leaving a naso-oral fistula. In this condition we have a very troublesome complication, making it necessary for the patient to keep the opening packed constantly, requiring removal after meals and withal leaving the mouth in a very unsanitary condition.

The underlying factor in nearly all of these cases is syphilis and, after the constitutional treatment has been pushed until all granulating surfaces have repaired, it is very desirable to close the opening permanently.

OPERATION AND CASE REPORTS.

An operation which I have done in several instances and which has accomplished this purpose may be described as follows: Assuming that the labial gingival structures are completely destroyed and that the lingual periosteum and mucous membrane extend well down to the normal line, two incisions are made through the lateral structure, either with scissors or knife, back up to the orifice of the fistula and far enough back on the two sides to make the tongue wide enough to cover the opening. The margin of the flap thus made is freshened and the corners made round. The next step is to freshen the orifice of the fistula, denuding well the internal surface of the lip for a distance equal to the thickness of the flap. The flap is now turned up over the orifice of the fistula and sutured there with silkworm gut. In the three cases in which the operation has been performed (two of which are described below) the results have been perfectly satisfactory.

Case 1.—*Patient.*—A man, aged 50, with infection of the mandible. He had lost all of his lower teeth but three, and the cavity included practically all of the mandible on its external surface, the bone being bare throughout. The alveolar process, on its external margin, including the cavities left by the extracted teeth, stood out perfectly nude in the floor of the mouth. It had required just a month for the case to advance to the condition described.

*Operation.*—This included a complete removal of the external half of the mandible from the second molar on the right side to the second bicuspid on the left, through the roots of the teeth and to external inferior margin of the bone. This left the internal alveolar plate intact throughout with the periosteum undisturbed. The cavity was mopped out with pure tincture of iodin. The usual method of procedure would doubtless have been to pack the entire cavity, with the hope that the bone would heal by being granulated over from later approximation of the external periosteum. It was decided, however, that such an extensive cavity should be immediately obliterated. Dependent drainage was absolutely necessary if this was to be accomplished; hence an incision was made from the lowest point of the cavity in the median line through the skin under the chin large enough to admit a rubber drain the size of a lead pencil. The next step was to stitch together with catgut the labial and buccal gingival margins, thus closing off the field of operation entirely from the oral cavity.

*Postoperative History.*—To my intense gratification the two gingival margins did completely unite and not a drop of pus was ever found in the oral cavity. The drainage established from below was quite sufficient to carry off the small quantity of reparative lymph and detritus and the patient was practically well in ten days after his operation.

CASE 2.—*Patient.*—A man, aged 30, had extensive infection of the mandible beginning from an infected tooth. Seven or eight teeth had been extracted and the entire external surface of the mandible was bare. The oral cavity was welling full of pus from the extensive suppurating area. The disease had developed rapidly and was running an acute course.

*Operation.*—This was practically the same as that performed on the above case. After the removal of the external half of the bone and disinfection of the wound, drainage was established through the skin under the chin. The gingival margins were approximated by catgut sutures.

*Postoperative History.*—The openings did not unite throughout as in the former case; two or three openings communicated with the mouth. This case was no doubt syphilitic and constitutional treatment was administered before the disease could be controlled. It was several months before the system was impressed with the medication and the wound closed.

CONCLUSIONS

1. Dentists should realize the seriousness of the most frequent operation they perform, namely, that of devitalizing and extracting pulps, since infection and serious bone destruction originate from this cause.

2. Destruction of the bony floor of the antrum does not necessarily mean perforation of the membranous floor or infection.

3. An alveolar fistula leading into a cavity where a considerable portion of a tooth is exposed requires extraction of this tooth before complete recovery can be expected.

4. Persistent headaches and general reduction in health are frequently caused by very insidious alveolar abscesses.

5. In destruction of the mandible, requiring removal of bone, it is advisable to establish drainage through the chin and approximate the gingival margins with sutures so as to shut off a pus cavity from the oral cavity.

6. Naso-oral fistula may be closed by a membranous flap from the roof of the mouth.

7. In all suppurative conditions of the mouth pure tincture of iodin should be used as a disinfectant.

### ABSTRACT OF DISCUSSION.

Dr. William C. Fisher, New York: In this synopsis a statement is made that probably one-half the cases of necrosis of the maxillary bones are due to syphilis. I think probably one thing is lost sight of, and that is that the administration of mercury for syphilis very often starts up symptoms for which the disease is blamed. We can get the very same symptoms from the administration of mercury that are caused by syphilis.

Dr. James E. Power, Providence, R. I.: To me it seems unnecessary to make an incision such as described by the essayist. As I understand the operation, the doctor draws the flap across the opening of the top, within the oral cavity, and then makes an incision below and on the external surface for drainage. I do not think that one is justified in making an external incision on the face, and surely not in a case like the one described. I do not quite see any disadvantage in proper drainage through the mouth, even in view of the fact that there may be bacteria present which are capable of causing trouble in the alimentary canal. With the external incision, even if he gets union within the oral cavity by first intention, some days will elapse before he gets a sufficiently strong union to prevent back drainage. I believe that the term syphilis is getting to be used in the medical profession about the same as neuralgia in the dental profession. It seems that about every condition which is beyond the diagnostic capabilities of the practitioner is pronounced syphilis. I am conscious of the prevalence of syphilis, but I believe that many mistakes are made. From my experience I would not care to accept the statement in the matter as final, that over one-half the cases of necrosis of the superior maxilla were due to syphilitic conditions. A good many times the argument for the diagnosis of syphilis is simply that the condition improved under potassium iodid. We all know that many conditions will improve under potassium iodid. Without a good history the only true test is the Wassermann reaction, and that is very seldom resorted to by the general practitioner because it is a complex process and requires the skill of a laboratory expert. Dr. Fisher says that he believes that many of the cases of para-syphilitic conditions are due to the drugs. I believe, however, that all conditions that come from treated syphilis are from the disease and not from the drug. We might get a profuse flow of saliva from too much mercury, but I do not believe that we get necrosis of bone, gummata and other pathologic manifestations which are associated with syphilis and which are erroneously attributed to the use of the drug. I believe 100 of the para-syphilitic conditions are due to the disease where but one is due to mercury. My authority for this statement is Dr. Charles M. Whitney, of Boston. I am speaking of cases properly treated. Of course, if a practitioner continues to use mercury after symptoms show that the drug should be suspended he is likely to be confronted with serious conditions. Salivation was used as the indicator in the early days of the treatment of this disease, but that day has passed. They are treating syphilis more scientifically to-day. It is seldom, if ever, that a syphilologist will permit a patient to reach the stage of profuse salivation.

Dr. Eugene Talbot: Most individuals who have alveolar abscesses are run down. In case of syphilis, mercurial, or any other drug poisonings, there is a want of tonicity of the nervous system, in all cases, we may say, due to irritation. When irritation takes place the peri-dental membrane becomes involved and inflammation is set up, which causes pressure and pain around the root of the tooth. With this pressure, inflammation is set up in the arteries of the bone, the Haversian canals and in the vessels of von Ebner. That inflammation causes absorption of the bone proper, leaving the fibrous tissue (trabeculae). This might be likened to the plastering on the side of a building. If it were not for the lathing the plastering would not stick. If the lathing (trabeculae) be removed, then the alveolar tissue or bone cannot be restored. The extent of formation of this abscess depends entirely on the amount of irritation and also on the weakened condition of the patient. It may go on, extending down into the bone tissue through these canals, and the bone proper (that is, the bone-cells which are held together by fibrous tissue) is absorbed, leaving the fibrous tissue intact. This fibrous tissue becomes organized and forms the sac of the abscess. The inflammation extends through these Haversian canals and produces absorption outward, just as when pebbles are thrown into a small lake, the waves enlarge and spread outward, and if you throw several pebbles into the lake at different localities these waves extend until they coalesce. The principle of bone absorption is the same. Absorption goes on around each Haversian canal until the entire area is involved, and we not only have absorption going on in the Haversian canals, but the vessels of von Ebner become involved.

These are not so large as the Haversian canals, so the process of absorption takes longer, but the principle is the same. It burrows right through the bone

Dr. Frederick Noyes, Chicago: The absorption of bone under the conditions just stated may be considered as a response to a mechanical condition of pressure, which is the result of the exudate which comes from the inflammation, and the absorption occurs in all the spaces of the bone in the neighborhood of the inflammation.

Dr. C. W. Harned: I think anyone who has made a practice of closing wounds to prevent secretions of the mouth getting into them will never give it up. I never fail to close up a cavity if possible, after removing all necrotic tissue. I think such wounds will seal in 36 hours, so that no saliva will get into the wound. Pus is rarely present after all the necrotic tissue has been removed. I want to commend the essayist for making this point in the matter of drainage.

Dr. James E. Power: The last speaker states that in his antral diseases he cleans the antrum and then closes the opening through which he operated. In all the antral cases which I have treated I have been obliged to follow the currettage treatment with irrigation. I would like to know how he irrigates if he closes the opening, or what procedure he follows.

Dr. C. W. Harned: I prefer going in above the bicuspids when I think disease involves the antrum. Thorough ventilation as well as drainage through the nose on that side is important. I make an opening through the internal wall of the antral cavity into the meatus, after the antrum is cleaned. Close up the mucous membrane on the outside after you have packed. Remove packing the next day through the nostril. Always close mucous membrane on the outside immediately, and irrigate as little as possible. I find that when I irrigate the least the patients get along the best. If all the pus and necrotic tissue is removed, and the cavity sterilized, very little irrigation will be found necessary.—*Journal A. M. A.*, October 8th, 1910.

